# The relationship between severity of drug problems and perceived interdependence of drug use and sexual intercourse among adult males in drug addiction rehabilitation centers in Japan

**DOI:** 10.1186/s13011-020-00339-6

**Published:** 2021-01-07

**Authors:** Risa Yamada, Takuya Shimane, Ayumi Kondo, Masako Yonezawa, Toshihiko Matsumoto

**Affiliations:** Department of Drug Dependence Research, National Institute of Mental Health, National Center of Neurology and Psychiatry, 4-1-1 Ogawa-Higashi, Kodaira, Tokyo, 187-8553 Japan

**Keywords:** Drug use, Sexual behaviors, Relapse prevention, Sexually transmitted diseases, Methamphetamine

## Abstract

**Background:**

Consuming drugs in conjunction with sexual intercourse may shape the perceived interdependence of drug use and sexual intercourse (PIDS). Additionally, the severity of drug problems may have a significant impact on PIDS. However, this relationship remains unverified. Therefore, this study investigates whether the severity of drug problems is associated with PIDS among adult males in drug addiction rehabilitation centers (DARC) in Japan.

**Methods:**

This study was a secondary analysis of the “DARC Follow-Up Study in Japan” conducted by the National Center of Neurology and Psychiatry in 2016, in which participants from 46 facilities completed a self-report questionnaire. A total of 440 males with drug dependence were included in the analysis. We analyzed participants’ demographic characteristics, history of sexually transmitted disease diagnoses, and responses to questions related to drug use (e.g., primary drug use and PIDS). Additionally, we measured the severity of drug problems using the Japanese version of the Drug Abuse Screening Test-20 (DAST-20).

**Results:**

The median age of the participants was 42 years. The median DAST-20 score was 14.0, the primary drug was methamphetamine (61.4%) and new psychoactive substances (NPS: 13.6%). Multivariate analysis indicated that participants’ experiences with unprotected sexual intercourse (“mostly a non-condom user”: adjusted odds ratio (AOR) = 4.410), methamphetamine use (AOR = 3.220), new psychoactive substances use (AOR = 2.744), and the DAST-20 score (AOR = 1.093) were associated with PIDS.

**Conclusions:**

This study indicated that the frequency of unprotected sexual intercourse under the influence of drugs, methamphetamine and NPS use were strongly associated with PIDS. The severity of drug problems was also significantly associated with PIDS. It is necessary to develop culturally appropriate treatment programs adapted to the needs of patients who experience strong PIDS.

**Supplementary Information:**

The online version contains supplementary material available at 10.1186/s13011-020-00339-6.

## Background

The use of illegal drugs is a significant concern worldwide. In 2018, 5.3% of the world’s population between the ages of 15 and 64 years had used illegal drugs [1]. The use of illegal drugs within the general Japanese population is not as serious as it is in other countries; a 2015 nationwide survey of the general population reported that only 0.1% of respondents had used an illegal drug in the previous 12 months [2].

However, a survey conducted among rave-attending populations in Japan [3] reported a lifetime prevalence of marijuana use of 22.4%, whereas the prevalence of 3,4-methylenedioxymethamphetamine (MDMA) use was 8.0%. According to internet surveys answered by men who have sex with men (MSM) in Japan [4], 65.5% of respondents had used illegal drugs in their lifetime. As overseas research indicates, MSM and rave attenders are at an especially high risk of drug use [5–7], with this likely also being true in the Japanese context.

People who use drugs tend to engage in risky sexual behaviors (e.g., sexual intercourse without a condom and having multiple sex partners). A study of senior high school students showed that illegal drug use was associated with engaging in unprotected sex (odds ratio (OR) = 2.44, *p* < 0.05) [8]. In a young adult population survey, males classified into the high-risk sexual behavior group (those who had experienced unprotected sex and multiple sexual partners within the previous year) had a higher incidence of unprotected sex in conjunction with alcohol and drug use than males classified into the low-risk sexual behavior group (54.3% vs. 5.5%, *p* < 0.001) [9]. A study of male commercial sex workers (CSWs) found that male CSWs who used drugs had a lower proportion of consistent condom use with female clients than male CSWs who did not use drugs (28.0% vs. 81.2%, *p* < 0.001) [10]. Research on the association between drug use and risky sexual behaviors is quite limited in Japan. A study on MSM reported that individuals who had used illegal drugs exhibited a higher likelihood of engaging in unprotected anal sexual intercourse (22.1% vs. 57.0%, *p* < 0.001) and having more than six sexual partners (19.7% vs. 33.1%, *p* < 0.001) than those who did not use illegal drugs [4].

Furthermore, drug problems are associated with risky sexual behaviors. A survey on stimulant users (e.g., those who used amphetamines or MDMA) conducted in drug detoxification and rehabilitation centers revealed that a history of severe acute intoxication after drug use during the previous year (adjusted odds ratio (AOR) = 2.11, *p* < 0.05), as well as a longer duration of drug use during their lifetime (AOR = 1.76, *p* < 0.05) was associated with having multiple sex partners during the previous year. Moreover, usage of multiple drugs during the previous year was associated with a higher risk of having unprotected sex during the previous year (AOR = 1.55, *p* < 0.05) [11].

These previous findings suggest that consuming drugs in conjunction with sexual intercourse leads individuals to associate drug use with engagement in risky sexual activities, reflecting a potentially strong perceived interdependence of drug use and sexual intercourse (PIDS). When people who use drugs engage in sexual intercourse, it may lead to drug craving, which may, in turn, prompt hypersexuality via the mesolimbic dopamine reward pathway [12]. The prevalence of strong PIDS may increase the severity of an individual’s drug problems, including the risk of sexually transmitted diseases (STDs) and unwanted pregnancies. In a self-report study of individuals receiving treatment for drug dependence in the United States, those who had used methamphetamine or cocaine, in contrast to those who had used opioids, reported strong PIDS; further, treatment was needed to separate drug use and sexual intercourse [13]. The previous study showed that PIDS have highlighted its association with psychoactive substances; however, other related factors have not been adequately explored. Among persons for whom the severity of drug problems is relatively high, PIDS is assumed to be stronger. However, the exact nature of this relationship remains unverified.

In this study, we hypothesize that the severity of drug problems is associated with an individual’s PIDS. Thus, we investigate the severity of drug problems, using the Japanese version of the Drug Abuse Screening Test-20 (DAST-20), and PIDS among adult males in drug addiction rehabilitation centers (DARC) in Japan. Elucidating these relationships, it would help design and validate treatment plans for individuals among whom strong PIDS has been identified.

## Methods

### Participants and procedure

This study is a secondary analysis of the “DARC Follow-Up Study in Japan” conducted by our team at the National Center of Neurology and Psychiatry (NCNP) in 2016 [14]. DARC, which was founded in 1985, is operated by staff who have experienced problems with drug use and have recovered at these facilities. There are approximately 60 DARC facilities across Japan. The participants of this study frequently attend meetings based on the 12-step Narcotics Anonymous program to recover from drug dependence; those who recovered serve as staff and assist others in their recovery process.

We visited rehabilitation centers from July to September 2016 and explained the significance and purpose of the study to the facility managers or full-time staff members. For this cross-sectional study, we sent manuals, consent forms, and questionnaires from October to December 2016 to 46 out of 57 rehabilitation facilities (80.7%) that agreed to participate. The facility managers or full-time staff clearly explained the study purpose to facility users, who could participate only after the provision of written informed consent. Confirmed participants were then required to answer a self-administered questionnaire. These documents were collected and mailed from each facility to the NCNP.

**Fig. 1:**
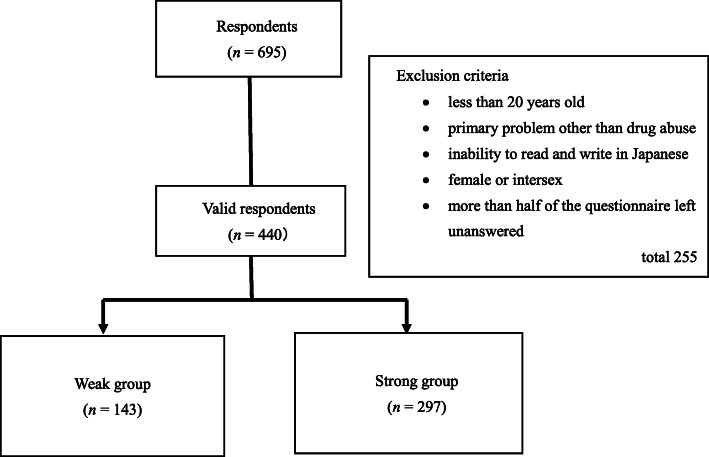
Flowchart of this study

After excluding the data of 255 participants based on the following criteria—(1) aged 19 years or younger, (2) primary problem other than drug use, (3) inability to read and write in Japanese, (4) female or intersex, and (5) more than half of the questionnaire left unanswered—we included 440 participants in the analysis (Fig. 1).

The analysis focused only on male participants for the following reasons: 1) More than 90% of the DARC participants in the primary survey were males. 2) This study considered not only the situation of using condoms in conjunction with drug use but also male initiatives for using condoms in conjunction with drug use. Male condoms are widely popular. Therefore, using condoms are generally determined by male initiatives, and females tend to have passive roles. Thus, the response to the questionnaire item regarding the female condom use could be qualitatively different from males, making the interpretation of the results difficult. 3) There was only one intersex participant.

### Assessments of clinical and background information

In the current study, we analyzed the participants’ demographic characteristics and history of STD diagnoses as well as drug use-related responses to questionnaires such as the Japanese version of the DAST-20 administered in the initial study.

Demographic characteristics included age (20–74 years), sexual orientation (heterosexual or not), education (less than high school or completed high school), employment status (employed or not), living conditions, and current chronic physical diseases. Self-reported STD diagnosis included history of hepatitis A, B, and C, chlamydia, gonorrhea, syphilis, and human immunodeficiency virus (HIV).

We included questions related to drug use, such as the experience of injecting and sharing needles, and history of treatment and crime. The following were the reported primary drugs of dependence: organic solvent, marijuana, methamphetamine, cocaine, heroin, MDMA, new psychoactive substances (NPS), prescription drugs (i.e., only for the purpose of misuse), and over-the-counter (OTC) drugs, such as analgesics (i.e., only for the purpose of misuse). Participants were only able to select one option for their primary drug of dependence.

We included a question on sexual behavior, which assessed the experience of unprotected sex under the influence of drugs using a three-point Likert scale (1 = *always use condoms*, 2 = *sometimes a non-condom user*, and 3 = *mostly a non-condom user*).

A questionnaire item on PIDS was included in this study. No reliable and validated scale for measuring PIDS has been established as a survey instrument. However, PIDS could play a crucial role in understanding drug problems and problematic sexual activities. For example, Rawson et al. [13] reported that methamphetamine and cocaine use had a higher proportion of treatment seeking for PIDS than opiate and alcohol use. Therefore, our study analyzed this variable, the data for which was gathered using the following question “How strong is the relationship between drug use and sexual intercourse for you?”—This was answered on a four-point Likert scale (1 = extremely strong, 2 = strong, 3 = weak and 4 = extremely weak). The question wording was selected so as not to offend the participant, since drug use and sexual issues are sensitive subjects in Japan. Since, this item has not been previously tested for reliability and validity, caution is required in interpreting the results.

The Japanese version of the DAST-20 [15] is a reliable and valid tool for measuring the severity of drug dependence. The DAST-20 was developed by Skinner [16] as a screening tool to calculate the severity of drug problems through 20 yes/no questions. The DAST-20 score has a high probability of meeting the diagnostic criteria for drug dependence in the Diagnostic and Statistical Manual of Mental Disorders [17]. Moreover, the DAST-20 has five sub-factors (i.e., Dependence, Social Problems, Medical Problems, Poly-Drug use, and Previous Treatment) that evaluate drug problems from multiple perspectives. In this study, participants self-reported their answers [15]. Additionally, to make the measurement standards uniform, participants were asked to recall their drug use in the 12 months prior to joining DARC.

### Statistical analysis

We analyzed the data of 440 participants. First, we divided them into two groups based on their perception of the relationship between drug use and sexual intercourse. One group consisted of respondents whose PIDS was weak (1, extremely weak; 2, weak), while the other consisted of respondents whose PIDS was strong (3, strong; 4, extremely strong). We used Fisher’s exact test and a Mann–Whitney U test to compare the demographic and clinical characteristics of the participants in the two groups. Second, we used multiple logistic regression to calculate AORs and 95% confidence intervals (CIs) after simultaneously adjusting for potential confounders. Likewise, we categorized PIDS into weak and strong. Each variable included demographic characteristics (e.g., education, employment, and sexual orientation), primary drug of dependence, experience of injecting and sharing needles, treatment history for substance use disorders, drug-related criminal history, and unprotected sexual behavior. We analyzed candidate variables using Spearman rank correlations; no strong correlations were found (− 0.345 ≤ r ≤ 0.444). Based on these results, we assumed that no multicollinearity problems existed. Candidate variables using Spearman rank correlations showed in supplementary Table 1.

The threshold for statistical significance was set at *p* < 0.01. All statistical analyses were performed using SPSS software version 25.0 (SPSS Japan, Inc.).

## Results

The demographic and clinical characteristics of the participants in the two groups are shown in Table 1. The median age of the participants was 42 years, 85.9% were heterosexual, 43.9% had completed high school, 73.9% reported being unemployed, and 77.0% lived in a dormitory. When comparing the demographic and clinical characteristics of the two groups, age was significantly higher in the strong group than in the weak group (*p* = 0.008).

**Table 1:** Demographic and clinical characteristics of the participants in two groups

	perceived interdependence of sexual intercourse and drug use							
	weak group^a^		strong group^b^		Total		Analysis	
	*n* = 143		*n* = 297		*n* = 440		Statistic	*p*-value*
Age (median, Q1-Q3)	39	(32–46)	42	(36–48)	42	(34–47)	U = 24,555.000	**0.008**
Heterosexual: n (%)	127	(88.8)	251	(84.5)	378	(85.9)		0.245
Completed high school: n (%)	67	(46.9)	126	(42.4)	193	(43.9)		0.412
Unemployed: n (%)	111	(77.6)	214	(72.1)	325	(73.9)		0.247
Living condition: n (%)								
Living in a dormitory (rehabilitation center)	117	(81.8)	222	(74.7)	339	(77.0)		0.116
Daycare	11	(7.7)	30	(10.1)	41	(9.3)		0.486
Undergoing training as rehabilitation staff	15	(10.5)	45	(15.2)	60	(13.6)		0.235
Chronic disease (physical disease): n (%)	22	(15.4)	74	(25.2)	96	(22.0)		0.026
Primary drug: n (%)								
Methamphetamine	56	(39.2)	214	(72.1)	270	(61.4)		**< 0.001**
NPS	22	(15.4)	38	(12.8)	60	(13.6)		0.461
Organic solvent	14	(9.8)	12	(4.0)	26	(5.9)		0.029
Marijuana	11	(7.7)	13	(4.4)	24	(5.5)		0.179
Prescription drugs	17	(11.9)	7	(2.4)	24	(5.5)		**< 0.001**
Over-the-counter drugs	14	(9.8)	6	(2.0)	20	(4.5)		**0.001**
Others (cocaine, heroin, MDMA, gas)	9	(6.3)	7	(2.4)	16	(3.6)		0.055
Drug-related criminal history: n (%)	40	(28.0)	163	(54.9)	203	(46.1)		**< 0.001**
Never experienced treatment: n (%)	22	(15.5)	64	(21.8)	86	(19.7)		0.157
Consistent condom use under the influence of drugs: n (%)								
Always uses condoms	66	(46.2)	38	(12.8)	104	(23.7)		**< 0.001**
Sometimes a non-condom user	27	(18.9)	50	(16.9)	77	(17.5)		0.595
Mostly a non-condom user	50	(35.0)	208	(70.3)	258	(58.8)		**< 0.001**
Injecting and sharing needles during drug use: n (%)								
None	74	(52.9)	60	(20.5)	134	(31.0)		**< 0.001**
Only experienced injection	16	(11.4)	46	(15.8)	62	(14.4)		0.245
Experienced injection and sharing needles	50	(35.7)	186	(63.7)	236	(54.6)		**< 0.001**
Diagnosis of sexually transmitted disease in lifetime: n (%)								
Hepatitis A	0	(0.0)	1	(0.3)	1	(0.2)		1.000
Hepatitis B	1	(0.7)	18	(6.3)	19	(4.5)		**0.006**
Hepatitis C	19	(13.6)	92	(32.2)	111	(26.1)		**< 0.001**
Gonorrhea	6	(4.3)	30	(10.5)	36	(8.5)		0.040
Chlamydia	7	(5.0)	22	(7.7)	29	(6.8)		0.413
Syphilis	2	(1.4)	19	(6.6)	21	(4.9)		0.017
HIV	0	(0.0)	18	(6.3)	18	(4.2)		**0.001**
Never diagnosis of STD	110	(78.6)	139	(48.6)	249	(58.5)		**< 0.001**
DAST-20 total score (median, Q1-Q3)	13.0	(10–16)	15.0	(12–17)	14.0	(12–16)	U = 25,873.500	**< 0.001**

*p-value for Fisher’s exact test or Mann–Whitney U test

Significant *p*-values are shown in bold type and underlined (*p* < 0.01)

^a^ Weak group consisted of respondents who considered the relationship between drug use and sexual intercourse was perceived “extremely weak + weak”

^b^ Strong group consisted of respondents who considered the relationship between drug use and sexual intercourse was perceived “extremely strong + strong”

*Abbreviations*: *NPS* new psychoactive substances, *MDMA* 3, 4-methylenedioxymethamphetamine, *STDs* sexually transmitted diseases, *HIV* human immunodeficiency virus, *DAST* the Drug Abuse Screening Test

Drug characteristics and STDs for the two groups are shown in Table 1. The primary drug was methamphetamine (61.4%), followed by NPS (13.6%), organic solvent (5.9%), marijuana (5.5%), prescription drugs (5.5%), and OTC drugs (4.5%). Of the participants, 46.1% reported that they had no drug-related criminal history, and 54.6% had experience of injecting and sharing needles. The most common response to the question on unprotected sexual intercourse when under the influence of drugs was “mostly a non-condom user” (58.8%). The most diagnosed STD was hepatitis C (26.1%), followed by gonorrhea (8.5%) and chlamydia (6.8%). Additionally, the median DAST-20 score was 14.0.

The strong group reported a significantly higher proportional dependence on methamphetamine as a primary drug than the weak group (*p* < 0.001). However, the weak group reported significantly higher proportional dependence on prescription and OTC drugs as primary drugs than the strong group (*p* < 0.001 and *p* = 0.001, respectively). Moreover, regarding the experience of unprotected sex when under the influence of drugs, the proportion of “mostly a non-condom user” response was significantly higher in the strong group than in the weak group (*p* < 0.001). Regarding the diagnosis of STDs, the prevalence of hepatitis B, hepatitis C, and HIV was significantly higher in the strong group than in the weak group (*p* = 0.006, *p* < 0.001, and *p* = 0.001, respectively). Additionally, the DAST-20 scores were significantly higher in the strong group than in the weak group (*p* < 0.001).

### Multiple logistic regression models

Table 2 shows multivariate AORs for PIDS. A total of 143 respondents (32.5%) perceived interdependence of drug use and sexual intercourse as weak (1, extremely weak; 2, weak), while 297 (67.5%) perceived it as strong (3, strong; 4, extremely strong). In the multivariate analysis, “mostly a non-condom user” response (AOR = 4.410; 95% CI = 2.444–7.959), methamphetamine use (AOR = 3.220; 95% CI = 1.723–6.017), NPS use (AOR = 2.744; 95% CI = 1.292–5.826), “sometimes a non-condom user” response (AOR = 2.536; 95% CI = 1.257–5.116), and DAST-20 score (AOR = 1.093; 95% CI = 1.029–1.160) were significantly associated with PIDS.

**Table 2:** Multivariate logistic regression analysis of perceived sex and drug interdependence

	perceived interdependence of sexual intercourse and drug use was strong^a^ ( = 1) and weak^b^ ( = 0)											
	Crude odds ratio	95% CI			Wald	*p*-value	Adjusted odds ratio	95% CI			Wald	*p*-value
Age	1.031	1.009	–	1.053	7.861	**0.005**	1.012	0.983	–	1.043	0.644	0.422
Education												
≧High school (ref)	1						1					
Junior high school	1.196	0.801	–	1.787	0.768	0.381	0.626	0.371	–	1.057	3.075	0.080
Sexual orientation												
Heterosexual (ref)	1						1					
Non-heterosexual	1.455	0.792	–	2.671	1.462	0.227	1.211	0.574	–	2.555	0.252	0.616
Employment												
Employed (ref)	1						1					
Unemployed	0.743	0.466	–	1.187	1.545	0.214	0.713	0.411	–	1.237	1.452	0.228
Drug-related criminal record												
None (ref)	1						1					
≧1	3.132	2.036	–	4.819	26.986	**< 0.001**	1.732	0.947	–	3.166	3.185	0.074
Chronic disease (Physical disease)												
None (ref)	1						1					
chronic disease	1.850	1.094	–	3.128	5273	0.022	0.856	0.436	–	1.679	0.205	0.651
The history of treatment												
None (ref)	1						1					
experienced treatment	0.659	0.387	–	1.122	2.360	0.124	0.847	0.449	–	1.597	0.263	0.608
The primary substance of dependence												
Methamphetamine	5.520	3.413	–	8.926	48.530	**< 0.001**	3.220	1.723	–	6.017	13.429	**< 0.001**
NPS	2.495	1.305	–	4.771	7.642	**0.006**	2.744	1.292	–	5.826	6.903	**0.009**
Other than methamphetamine and NPS (ref)	1						1					
Injecting and sharing needles during drug use												
Never experienced using needle (ref)	1						1					
Only experienced injecting	3.546	1.827	–	6.881	14.003	**< 0.001**	1.986	0.892	–	4.425	2.819	0.093
Experienced injecting and sharing needles	4.588	2.891	–	7.282	41.775	**< 0.001**	1.871	0.975	–	3.591	3.548	0.060
Consistent condom use under the influence of drugs												
Always uses condoms (ref)	1						1					
Sometimes a non-condom user	3.216	1.739	–	5.950	13.855	**< 0.001**	2.536	1.257	–	5.116	6.748	**0.009**
Mostly a non-condom user	7.225	4.362	–	11.967	59.009	**< 0.001**	4.410	2.444	–	7.959	24.277	**< 0.001**
DAST-20 score	1.104	1.050	–	1.161	14.874	**< 0.001**	1.093	1.029	–	1.160	8.348	**0.004**

Significant *p*-values are shown in bold type and underlined (*p* < 0.01)

Explanatory variable is relationship between drug use and sexual intercourse responded as strong^a^ ( = 1) and weak^b^ ( = 0)

a: Strong consisted of respondents who considered the relationship between drug use and sexual intercourse was perceived “extremely strong + strong”

b: Weak consisted of respondents who considered the relationship between drug use and sexual intercourse was perceived “extremely weak + weak”

*Abbreviations*: *NPS* new psychoactive substances, *DAST* the drug abuse screening test, *CI* confidence interval

## Discussions

This is the first study to examine the association between severity of drug problems and PIDS among adult males in DARC in Japan. As expected, methamphetamine as the primary drug of dependence was an important factor in PIDS, regardless of demographics, history of treatment for drug dependence, and drug-related history of criminal activity. This result is similar to that of previous research conducted in the United States [13]. Furthermore, the current study showed that NPS as the primary drug of dependence was also associated with PIDS, which has not been previously reported. Both methamphetamine and NPS are psychoactive substances. Methamphetamine has been reported to exert a strong central nervous system excitatory effect in addition to enhancing sexual desire and euphoria and inhibiting ejaculation [18]. Additionally, some NPS contain cathinone, which has the same psychoactive effect as methamphetamine [19]. Therefore, as reported in previous research, those who used NPS did not have a high condom usage rate [20]. Ultimately, psychoactive substances, such as methamphetamine and NPS, were associated with PIDS, as were risky sexual behaviors and enhanced sexual feelings and performance.

Furthermore, this study showed that the frequency of unprotected sexual intercourse under the influence of drugs was the most important factor (“mostly a non-condom user”: AOR = 4.410) in PIDS, which has not been previously reported. Therefore, it is necessary to determine treatment methods specifically focusing on problems related to PIDS for those who have unprotected sexual intercourse under the influence of drugs.

The primary hypothesis of this study was that the severity of drug problems, as revealed by the participants’ DAST-20 scores, would be associated with their PIDS; accordingly, we conducted a logistic regression analysis. The results showed that participants’ DAST-20 scores were significantly associated with PIDS, even after adjusting for the factors of frequency of unprotected sexual intercourse under the influence of drugs, methamphetamine and NPS use. While strong relationships were found between PIDS and “mostly a non-condom user” (AOR = 4.410), methamphetamine use (AOR = 3.220) and NPS use (AOR = 2.744), the effect size for the DAST-20 score was rather weak (AOR = 1.093). Strong PIDS was associated with a high frequency of unprotected sexual intercourse under the influence of drugs, methamphetamine and NPS were the primary drugs of choice; these represent significant risk factors that require treatment and interventions to address strong PIDS. Furthermore, although the effect was secondary, the DAST-20 score was also a significant risk factor.

It is important for those who have a strong PIDS to receive multiple treatments and participate in support groups to address their drug use. For example, DARC are some of the most important facilities for providing support in daily life for people with drug-related psychosocial problems. People who belong to DARC live in dormitories and attend regular meetings based on the 12-step Narcotics Anonymous program to recover from their drug dependency. Leading a healthy lifestyle and receiving support from both staff and peers who have experienced similar problems are considered important factors for reducing and managing drug use issues [21, 22]. Additionally, the Serigaya Methamphetamine Relapse Prevention Program (SMARPP) is the only insurance-covered psychosocial treatment program for substance use disorders in Japan [23]. The SMARPP was developed at the Kanagawa Psychiatric Center in 2006 as an outpatient treatment program with a focus on cognitive-behavioral therapy, which primarily targets addiction to stimulants [23]. The program is based on the Matrix Model, a treatment program developed by Dr. Gordon Alan Marlatt [24], which has been widely implemented in the west coast of the United States. It adopts the principles of cognitive-behavioral therapy for the prevention of drug use and utilizes motivational interviews for support, while emphasizing the importance of gaining knowledge concerning one’s addiction and the acquisition of specific coping skills [25].

The relapse prevention program for substance use disorders offered by SMARPP includes sessions related to drug use and sexual behaviors, in which participants learn that using drugs increases their chances of engaging in risky sexual intercourse (e.g., sexual intercourse without condoms), thereby increasing their likelihood of contracting STDs [24, 25]. However, as this program is only one of the many offered by SMARPP, it is considered inadequate as a treatment strategy for those with drug use and sexual problems. There are treatment programs in other countries that focus on reducing individuals’ engagement in unprotected sexual intercourse and multiple sex partners under the influence of drugs. One such treatment program, called Real Men Are Safe, focuses on men with drug use-related problems and who practice risky sexual behaviors [26]. This five-session program has been used in the United States. For example, in the session on STDs, participants learn that drug use increases the probability of engaging in risky sexual intercourse, which increases the individual’s likelihood of contracting STDs. Additionally, this session encourages participants to roleplay, including how to say “no” to their partner’s offer to use drugs during intercourse. However, participants who engage in sexual intercourse when under the influence of drugs may lack the ability or maturity to gently, but firmly, reject their partners’ advances. Thus, learning practical assertive skills to communicate one’s own opinions while respecting those of another during sexual intercourse not only reduces the frequency of drug use during intercourse but also reduces the risk of contracting STDs. Moreover, in the session that addresses triggers for drug use and risky sexual behaviors, participants learn that there is a high possibility of relapse as there is a strong relationship between these two behaviors. Additionally, this session utilizes peer discussions on triggers and solutions for situations regarding drug relapse relating to sexual intercourse. Recognizing the perceived interdependence of drug use and sexual intercourse, and thinking about how to deal with each trigger, may be effective in reducing individuals’ drug use problems. Furthermore, compared to the single session of HIV education offered as standard treatment in the United States, the five sessions of Real Men Are Safe have been reported to reduce the frequency of sexual intercourse without condom use. Psychological education related to drug use and sexual intercourse, as well as learning the tools for self-control together with peer group discussions, may be effective in reducing sexual intercourse without condom use.

In conclusion, programs focusing on individuals who experience strong PIDS would be more effective if they included several sessions addressing this specific topic. There is an urgent need to develop and validate Japan-specific treatment programs tailored for those who experience strong PIDS.

### Limitations

This study has some limitations. First, this study measured PIDS using a self-report questionnaire. This item measured the perceived relationship between drug use and sexual intercourse, rather than measuring an actual relationship. Therefore, these findings need to be interpreted with caution. Second, the results of this study may not be applicable to people who use drugs outside DARC. The living conditions and individual backgrounds of those outside DARC are very different from those inside DARC. The median DAST-20 score in this study was 14.0, which is a severe score for drug problems. Future research should investigate and verify our findings in medical and criminal institutions. Third, because there are differences among countries in terms of drug problems, the results of this study may not be generalizable to other nations. Future research needs to investigate the relationship between drug use and sexual intercourse with the severity of drug problems while considering country-specific characteristics and differences. Fourth, this was a cross-sectional study and, as such, we were not able to make causal inferences. Prospective studies are needed to clarify causality between the severity of drug problems and PIDS.

## Conclusions

This study investigated whether the severity of drug problems is associated with PIDS among adult males in DARC in Japan. This study found that the DAST-20 scores were associated with PIDS, although the effect size was small. Specifically, the effect size was smaller than that for “mostly a non-condom user,” methamphetamine use, and NPS use. It is necessary to develop and validate treatment programs tailored to the Japanese context, focusing on problems associated with drug use and sexual intercourse.

## Supplementary Information

**Additional file 1: Supplementary Table 1.** Correlation between candidate variables.

## Data Availability

In order to protect the confidentiality of study participants, the data are not available.

## Abbreviations

AOR: Adjusted odds ratio
CI: Confidence interval
DARC: Drug addiction rehabilitation centers
DAST-20: Drug abuse screening test-20
HIV: Human immunodeficiency virus
MDMA: 3,4-methylenedioxymethamphetamine
MSM: Men who have sex with men
NCNP: National Center of Neurology and Psychiatry
NPS: New psychoactive substances
OR: Odds ratio
PIDS: Perceived interdependence of drug use and sexual intercourse
STD: Sexually transmitted disease
