# Supplementary material for: The relationship between severity of drug problems and perceived interdependence of drug use and sexual intercourse among adult males in drug addiction rehabilitation centers in Japan
Source: Subst Abuse Treat Prev Policy. 2021 Jan 7;16:5. doi: 10.1186/s13011-020-00339-6 (PMC7791778; doi:10.1186/s13011-020-00339-6)
Supplement: Supplementary file 1 — Additional file 1: Supplementary Table 1. Correlation between candidate variables. [file 13011_2020_339_MOESM1_ESM.docx]

| Supplementary table 1. Correlation between candidate variables | | | |  |  |  |  |  |  |  |
| --- | --- | --- | --- | --- | --- | --- | --- | --- | --- | --- |
|  | Completed high school | Sexual orientation | Unemployed | Drug-related criminal history | Chronic disease (physical disease) | Never experienced treatment | Primary drug | Injecting and sharing needles during drug use | Consistent condom use under the influence of drugs | DAST-20 total score |
| Age | 0.156** | 0.093 | -0.067 | 0.398** | - 0.345** | 0.021 | 0.016 | 0.279** | 0.074 | -0.019 |
| Completed high school |  | -0.009 | -0.040 | 0.320** | -0.057 | 0.017 | 0.037 | 0.289** | 0.121 | 0.046 |
| Sexual orientation |  |  | -0.012 | 0.035 | - 0.189** | 0.030 | -0.006 | 0.033 | 0.086 | -0.043 |
| Unemployed |  |  |  | -0.077 | 0.045 | 0.037 | 0.036 | -0.009 | 0.013 | 0.085 |
| Drug-related criminal history |  |  |  |  | - 0.214** | 0.050 | - 0.010 | 0.444** | 0.202** | 0.084 |
| Chronic disease (physical disease) |  |  |  |  |  | -0.028 | 0.089 | - 0.215** | - 0.172** | -0.044 |
| Never experienced treatment |  |  |  |  |  |  | -0.074 | 0.036 | 0.138* | -0.074 |
| Primary drug |  |  |  |  |  |  |  | - 0.221** | - 0.196** | 0.055 |
| Injecting and sharing needles during drug use |  |  |  |  |  |  |  |  | 0.294** | 0.093 |
| Consistent condom use under the influence of drugs |  |  |  |  |  |  |  |  |  | 0.186** |
| Correlation coefficients represent Spearman's ρ | | |  |  |  |  |  |  |  |  |
| ** p* < 0.01 |  |  |  |  |  |  |  |  |  |  |
| *** p <* 0.001 |  |  |  |  |  |  |  |  |  |  |
